# Measured Versus Predicted Prosthesis–Patient Mismatch after TAVR in Sievers Type 1 BAV: Incidence, Determinants, and Outcomes From the AD-HOC Registry

**DOI:** 10.1161/CIRCINTERVENTIONS.125.015994

**Published:** 2026-05-12

**Authors:** Tommaso Fabris, Federico Arturi, Andrea Buono, Chiara de Biase, Michele Bellamoli, Andrea Zito, Antonio Mangieri, Nicholas Montarello, Giuliano Costa, Mesfer Alfadhel, Ofi Koren, Simone Fezzi, Barbara Bellini, Mauro Massussi, Andrea Scotti, Lin Bai, Giulia Costa, Alessandro Mazzapicchi, Enrico Giacomin, Riccardo Gorla, Karsten Hug, Carlo Briguori, Luca Bettari, Antonio Messina, Mauro Boiago, Matthias Renker, Mario Garcia Gomez, Massimo Napodano, Chiara Fraccaro, Luca Nai Fovino, Giulia Masiero, Francesco Cardaioli, Francesco Putortì, Andrea Panza, Maria Luisa De Rosa, Carlo Trani, Giulia Laterra, Alessia Latini, Dario Pellegrini, Alfonso Ielasi, Ady Orbach, Uri Landes, Tobias Rheude, Luca Testa, Ignacio Amat Santos, Francesco Saia, Luca Favero, Carlo Cernetti, Mao Chen, Marianna Adamo, Azeem Latib, Anna Sonia Petronio, Matteo Montorfano, Raj R. Makkar, Francesco Burzotta, Marco Barbanti, Daniel J. Blackman, Darren Mylotte, Ole De Backer, Didier Tchètchè, Diego Maffeo, Won-Keun Kim, Giuseppe Tarantini

**Affiliations:** 1 Department of Cardiac, Thoracic and Vascular Sciences and Public Health, University of Padua Medical School, Italy (T.F., F.A., M.N., C.F., L.N.F., G.M., F.C., F.P., A.P., G.T.).; 2 Valve Center, Fondazione Poliambulanza Istituto Ospedaliero, Brescia, Italy (A.B., M. Bellamoli, L. Bettari, A. Messina, M.L.D.R., D. Maffeo).; 3 Clinique Pasteur, Toulouse, France (C. de Biase, M. Boiago, D.T.).; 4 Department of Cardiovascular and Pulmonary Sciences, Catholic University of the Sacred Heart, Rome, Italy (A.Z., C.T., F.B.).; 5 Cardio Center, IRCCS Humanitas Research Hospital, Rozzano-Milan, Italy (A. Mangieri, A. Latini).; 6 The Heart Center, Rigshospitalet, Copenhagen University Hospital, Denmark (N.M., O.D.B.).; 7 U.O.C. Cardiologia, Centro Alte Specialità e Trapianti, P.O. G. Rodolico, A.O.U. Policlinico–V. Emanuele, Università di Catania, Italy (Giuliano Costa).; 8 Department of Cardiology, Leeds Teaching Hospitals NHS Trust, United Kingdom (M. Alfadhel, D.J.B.).; 9 Department of Cardiology, Smidt Heart Institute, Cedars-Sinai Medical Center, Los Angeles, CA (O.K., R.R.M.).; 10 Department of Cardiology, University Hospitals Galway, Ireland (S.F., D. Mylotte).; 11 School of Medicine, Vita-Salute San Raffaele University, Milan, Italy (B.B.).; 12 Civil Hospital and University of Brescia, Italy (M. Massussi, M. Adamo).; 13 Montefiore Medical Center, NY (A.S., A. Latib).; 14 Department of Cardiology, West China Hospital, Sichuan University, Chengdu (L. Bai, M.C.).; 15 Cardiac Catheterization Laboratory, University of Pisa and Azienda Ospedaliero-Universitaria Pisana, Italy (Giulia Costa, A.S.P.).; 16 Cardiology Unit, Cardiac Thoracic and Vascular Department, IRCCS Azienda Ospedaliero-Universitaria di Bologna, Italy (A. Mazzapicchi, F.S.).; 17 Cardiology Unit, Cardio-Neuro-Vascular Department, Ca’ Foncello Hospital Azienda N 2 Marca Trevigiana, Treviso, Italy (E.G., L.F., C.C.).; 18 Department of Cardiology, IRCCS Policlinico San Donato, San Donato Milanese, Italy (R.G., L.T.).; 19 Department of Cardiovascular Diseases, German Heart Center Munich, Technical University Munich (K.H., T.R.).; 20 Interventional Cardiology Unit, Mediterranea Cardiocentro, Naples, Italy (C. Briguori).; 21 Kerckhoff Heart Center, Bad Nauheim, Germany (M.R., W.-K.K.).; 22 CIBERCV, Division of Cardiology, Hospital Clínico Universitario de Valladolid, Spain (M.G.G., I.A.S.).; 23 Università degli Studi di Enna “Kore,” Italy (G.L., M. Barbanti).; 24 Division of Cardiology, IRCCS Hospital Galeazzi-Sant’Ambrogio, Milan, Italy (D.P., A.I.).; 25 Edith Wolfson Medical Center, Cardiology Department, Holon, Israel (A.O., U.L.).; 26 School of Medicine, Vita-Salute San Raffaele University, Milan, Italy (M. Montorfano).; 27 Interventional Cardiology Unit, IRCCS San Raffaele Scientific Institute, Milan, Italy (B.B.).; 28 Tel-Aviv University, Israel (A.O., U.L.).; 29 Interventional Cardiology Unit, IRCCS San Raffaele Scientific Institute, Milan, Italy (M. Montorfano).

**Keywords:** aortic valve stenosis, bicuspid aortic valve disease, computed tomography angiography, transcatheter aortic valve replacement

## Abstract

**BACKGROUND:**

Evidence regarding prosthesis–patient mismatch (PPM), measured (mPPM), and predicted (pPPM), after transcatheter aortic valve replacement in bicuspid aortic valve stenosis remains limited. This study sought to evaluate the incidence, predictors, and prognostic implications of mPPM and pPPM in patients with Sievers type 1 bicuspid aortic valve undergoing transcatheter aortic valve replacement.

**METHODS:**

The AD-HOC registry is a retrospective, multicenter study including 781 patients with severe aortic stenosis and bicuspid aortic valve treated with transcatheter aortic valve replacement between 2016 and 2023 across 24 centers. PPM was defined according to Valve Academic Research Consortium-3 criteria. The primary outcome was all-cause mortality.

**RESULTS:**

Moderate-to-severe mPPM was more frequent than pPPM (22% versus 8%; *P*<0.001). Balloon-expandable valves were independently associated with both mPPM and pPPM, while smaller valve size and supra-annular sizing predicted only pPPM. During a mean follow-up of 621±470 days, neither mPPM nor pPPM was associated with mortality in the overall cohort. Among patients with a small annulus (≤430 mm^2^; n=145), pPPM occurrence was significantly higher (19% versus 5.5%; *P*<0.001) and was associated with increased all-cause mortality, but not with cardiovascular mortality.

**CONCLUSIONS:**

In patients with Sievers type 1 bicuspid aortic valve undergoing transcatheter aortic valve replacement, pPPM occurred less frequently than mPPM and was predominantly driven by anatomic characteristics and sizing strategies. Although pPPM was associated with increased all-cause mortality among patients with small annuli, this association did not extend to cardiovascular mortality and should be considered hypothesis-generating. Further prospective investigations are warranted to better delineate the impact of anatomic constraints on clinical outcomes in this anatomically challenging subset.

**WHAT IS KNOWN**

- The clinical relevance of both predicted and measured patient-prosthesis mismatch (PPM) after transcatheter aortic valve replacement remains unknown.
- Comprehensive analyses on the occurrence and prognostic role of both measured PPM (mPPM) and predicted PPM (pPPM) after transcatheter aortic valve replacement in bicuspid aortic valve are lacking.

**WHAT THE STUDY ADDS**

- In patients undergoing transcatheter aortic valve replacement in raphe-type bicuspid aortic valve, pPPM is less frequent than mPPM.
- Balloon-expandable valve use is a predictor of mPPM and pPPM.
- Small valve size and supra-annular sizing are associated with a higher risk of pPPM, but not mPPM
- Neither mPPM nor pPPM was associated with mortality in the overall cohort.

Bicuspid aortic valve (BAV) represents the most prevalent congenital heart defect and constitutes a critical risk factor for severe aortic stenosis (AS), particularly among younger patient populations.^1,2^ Transcatheter aortic valve replacement (TAVR) has emerged as a well-established therapeutic option for severe AS, with indications now expanding to include younger and lower-risk cohorts.^3^ Despite inherent technical challenges, the frequency of TAVR procedures in patients with BAV continues to increase, driven by favorable mid-term outcomes comparable to those observed in tricuspid aortic valve (TAV) anatomies.^4–8^

Prominent anatomic features of BAV, such as extensive leaflet calcification, tapered annular morphology, and intentional downsizing of transcatheter heart valves (THVs) based on supra-annular measurements, have been highlighted as potential risk factors for prosthesis–patient mismatch (PPM). While PPM is firmly established as a prognostic indicator of adverse outcomes following surgical aortic valve replacement, existing evidence regarding its prognostic implications post-TAVR remains inconclusive.^9–12^ Notably, PPM assessment methods differ substantially between TAVR and surgical aortic valve replacement studies: measured effective orifice area (mEOA) predominates in TAVR populations, whereas predicted effective orifice area (pEOA), derived from bioprosthesis-specific parameters, is the standard in surgical aortic valve replacement. Variability in flow conditions, pressure recovery, measurement inaccuracies, and interobserver differences further challenge the reliability of mEOA, and consequently, the reported incidence of measured PPM (mPPM) following TAVR.^13–16^ To address these inconsistencies, the concepts of pEOA and predicted PPM (pPPM) have recently been introduced into TAVR research.^17–19^ However, current literature has not demonstrated a definitive association between pPPM and clinical outcomes following TAVR in patients with TAV anatomy.^11,17,20^

Importantly, data regarding the incidence and prognostic relevance of pPPM following TAVR in BAV patients are currently lacking. Therefore, this study aimed to investigate and compare the incidence, predictive factors, and prognostic impact of both mPPM and pPPM among patients with BAV undergoing TAVR.

## Methods

### Data Availability Statement

Data will not be available to others.

### Study Population

The AD-HOC (Characteristics, Sizing, and Outcomes of Stenotic Raphe-Type Bicuspid Aortic Valves Treated with Transcatheter Device Implantation) Registry is a retrospective, observational, international, multicenter study. This investigator-driven registry includes patients with severe AS and Sievers type 1 BAV undergoing TAVR between 2016 and 2023 across 24 tertiary centers. Detailed methodology for the registry has been published previously.^21,22^ TAVR indications were determined by local Heart Teams, and written informed consent was obtained from all patients. Device type and size, as well as procedural and postprocedural management, were left to the discretion of each center. Data collection adhered to local practices and institutional review board approvals. The study complied with the Declaration of Helsinki and received ethics committee approval at all participating sites.

### Data Collection and End Points Definition

Echocardiographic images were analyzed by local expert operators at each center in accordance with current guidelines.^23,24^ Low-gradient AS was defined by a preprocedural mean gradient <40 mm Hg. Multislice computed tomography scans were retrospectively reviewed by local experts, and detailed aortic root parameters were assessed according to established methodologies.^21^ Supra-annular sizing methods, including Bicuspid Aortic Valve Anatomy and Relationship with Devices, Level of Implantation at the Raphe, and Calcium Algorithm Sizing for Bicuspid Evaluation With Raphe, were applied to all patients.^25–27^ A tapered configuration was defined when at least 2 of these methods suggested potential benefit from intentional downsizing of the THV at the supra-annular level compared with conventional annular sizing.^28^ Patients were subsequently categorized into 2 groups: those sized according to conventional annular dimensions and those sized primarily using supra-annular measurements.

An annular area ≤430 mm^2^ defined the small-annulus subgroup.^29^ A commissural diameter ≤23 mm (except for the 29-mm Evolut platform, which exceeds 430 mm^2^) defined the small-THV category.^30^ pEOA values for each THV type and size were obtained from published literature^11,19^; valves without available reference EOA data were excluded. PPM was defined using the indexed EOA, adjusted for body mass index, according to Valve Academic Research Consortium-3 recommendations.^31^ Follow-up was performed according to local practice, and all clinical events were adjudicated per Valve Academic Research Consortium-3 criteria.^31^ Prediction and survival analyses focused on moderate-to-severe PPM. The primary outcome was all-cause mortality at the longest available follow-up; secondary outcomes included cardiovascular mortality, heart failure hospitalization, bioprosthetic valve dysfunction, and a composite of transient ischemic attack and stroke.

### Statistical Analysis

Categorical variables are presented as counts and percentages; comparisons between groups were performed using Fisher exact test or the χ^2^ test, as appropriate. Paired binary variables were analyzed using McNemar test. Distribution of continuous variables was assessed with the Shapiro-Wilk test. As almost all variables exhibited a non-normal distribution, they are presented as medians (interquartile range) for consistency. Comparison was performed using the Mann-Whitney *U* test or Kruskal-Wallis test, as appropriate. Paired continuous variables were compared using the Wilcoxon signed-rank test. Predictors of mPPM and pPPM were identified using logistic regression analyses. Procedural and postprocedural variables (eg, predilatation, paravalvular leak) were analyzed only for mPPM, as pPPM is independent of procedural outcomes. Being this the first study specifically evaluating predictors of PPM in this clinical setting, univariate screening was used as the primary variable selection strategy, and variables expressing a *P*<0.10 in univariate analysis were entered into multivariable models. Annular area was retained in all models for clinical relevance. Collinearity was assessed using Pearson correlation coefficients (r>0.7) and variance inflation factors (<2.5). Only 1 parameter per annular or supra-annular measurement was included in the final models. Time-dependent outcomes were evaluated using the Log-rank test and Cox regression, with results expressed as hazard ratios (HRs) and 95% CIs. Kaplan-Meier curves were used to illustrate event-free survival. Exploratory subgroup analyses were performed for patients with small annuli. Following the introduction of a fifth-generation balloon-expandable valve (BEV) incorporating updated leaflet technology and improved hemodynamic performance, a supplemental predictive analysis was performed; valve sizes implanted with earlier-generation BEV were re-modeled as fifth-generation devices, and observed rates of pPPM were compared with the projected rates for the current valve generation.^32^ For each analysis, an available-case approach was used, with no imputation technique used to deal with missing data. All analyses were performed using Jamovi v2.6.19.0. Statistical significance was defined as a 2-tailed *P*<0.05.

## Results

### Study Population

**Figure 1.**
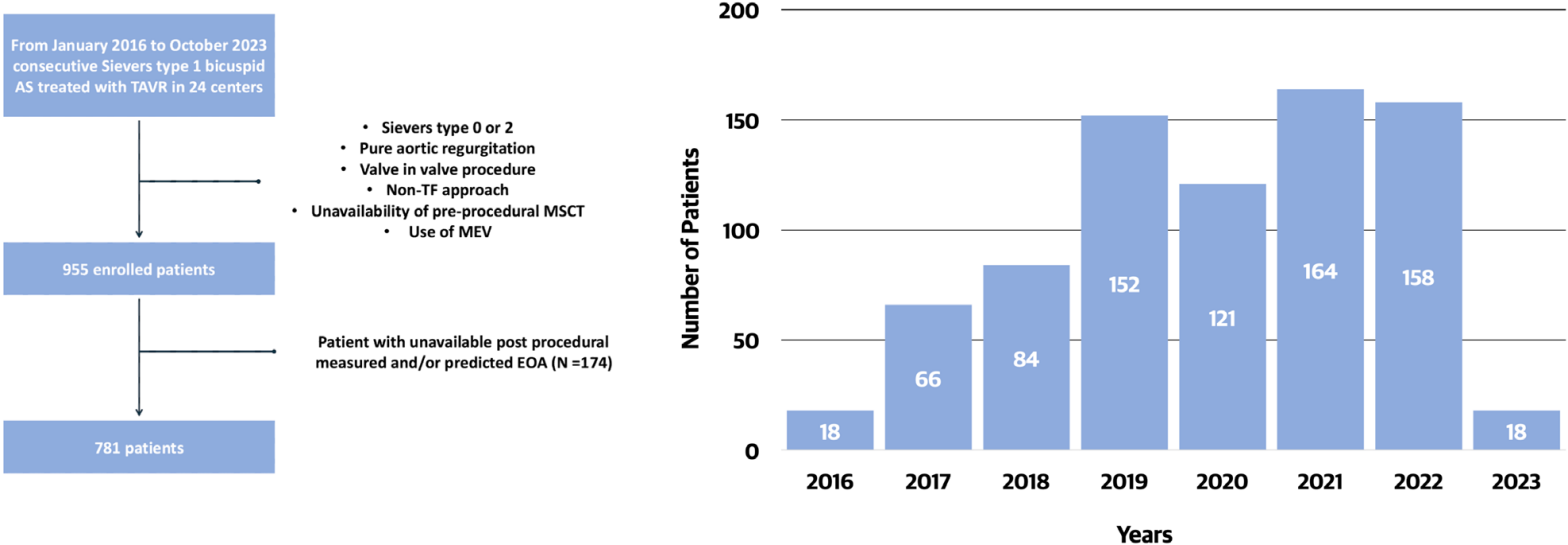
**Study flow-chart and enrollment period.** Study flow chart (**A**). Patient enrolled for each study’s year (**B**). AS indicates aortic stenosis; EOA, effective orifice area; MEV, mechanically expanded valve; MSCT, multislice computer tomography; TAVR, transcatheter aortic valve replacement; and TF, transfemoral.

A total of 955 consecutive patients with Sievers type 1 BAV anatomy underwent TAVR between January 2016 and October 2023 across 24 tertiary centers (Figure 1). Patients lacking available data on EOA or indexed EOA, as well as those who received THV without a predicted valve area reported in existing literature, were excluded from the analysis (Figure 1). Ultimately, 781 patients were included, of whom 496 (64%) were male (Figure 1; Table 1). The median patient age was 78 years (73–83 years), with a median Society of Thoracic Surgeons Predicted Risk of Mortality score of 2.40% (1.5%–3.6%). The most common raphe location was between the right and left coronary cusps (85%), and 314 patients (40%) presented with a tapered aortic root anatomy. Additional clinical and anatomic baseline characteristics are detailed in Table 1. BEV were implanted in 47%, and self-expanding valves (SEV) were used in 53%. Supra-annular THV sizing was employed in 161 patients (21%). Comprehensive procedural characteristics and device-specific details are presented in Table 2 and Table S1.

**Table 1.**
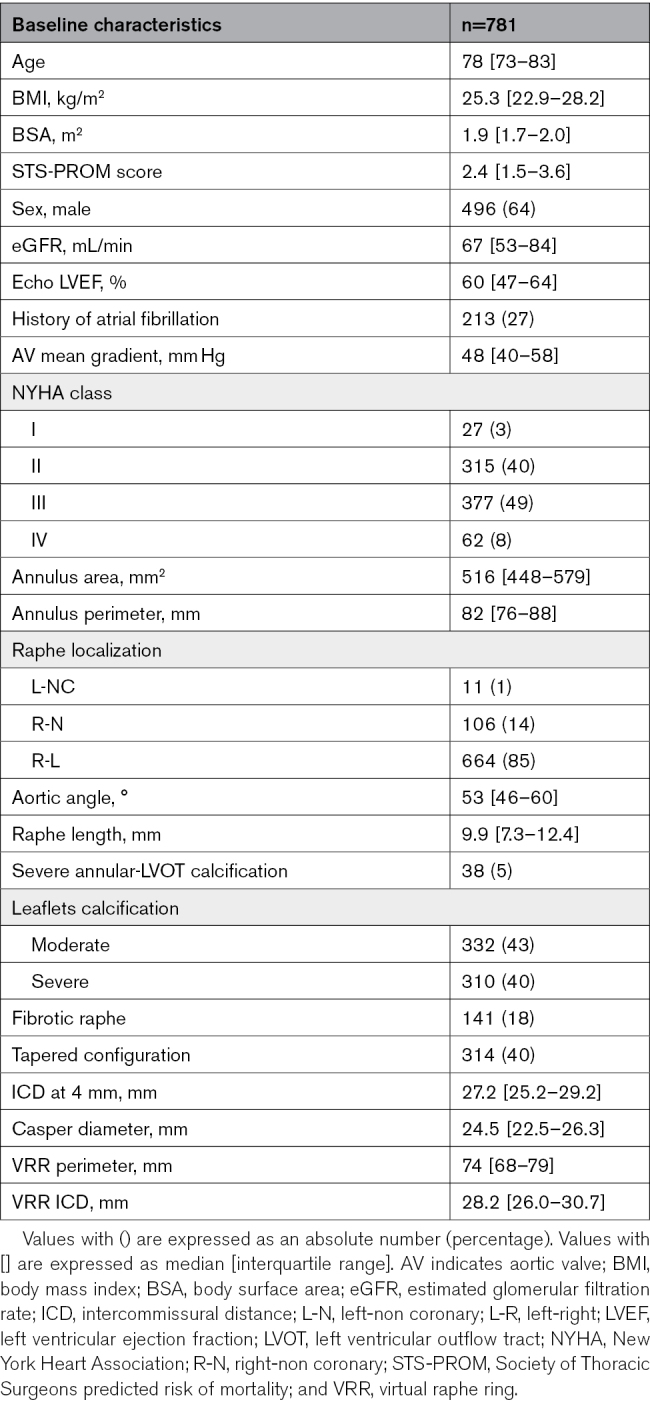
Baseline Characteristics of the Study Population

**Table 2.**
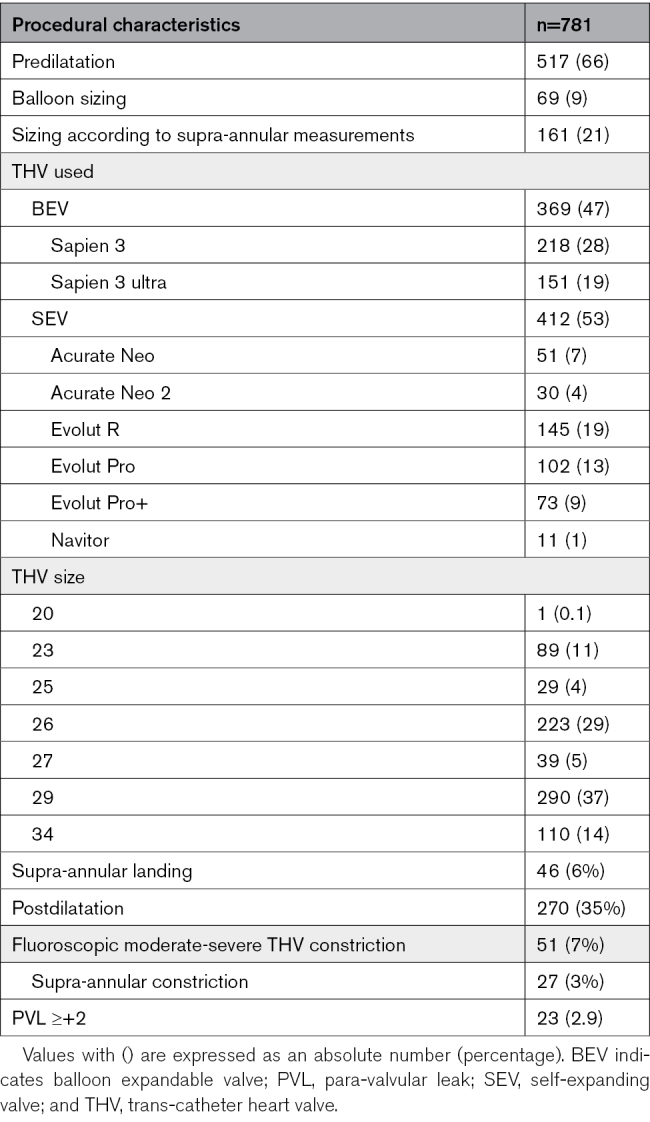
Procedural Characteristics of the Study Population

### Prevalence and Difference Between mPPM and pPPM

**Figure 2.**
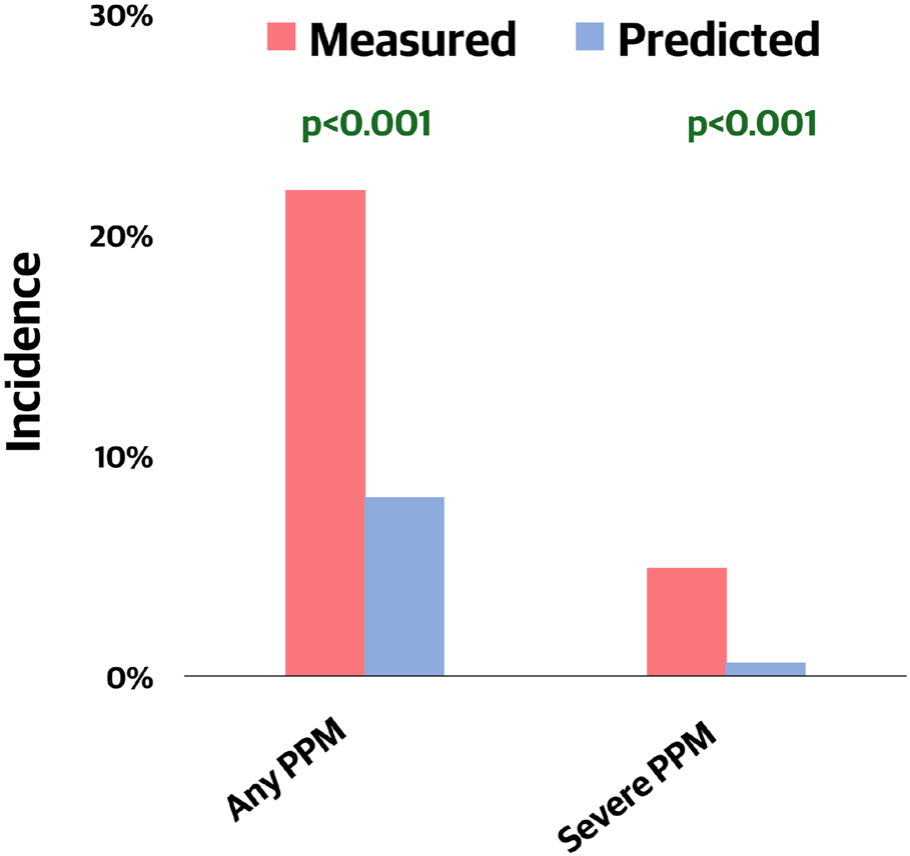
Incidence of measured and predicted prosthesis-patient mismatch (PPM).

On paired-sample analysis, mEOA and indexed EOA were marginally greater than predicted values, as shown in Table 2. Moderate-to-severe mPPM was significantly more common than pPPM (Table 3; Figure 2), and severe mPPM occurred more often than severe pPPM, which was confined to only 5 of 781 patients. Patients with moderate-to-severe mPPM exhibited higher postprocedural gradients (12 [9–17] mm Hg versus 8 [6–11] mm Hg; *P*<0.001) and left ventricular ejection fraction (60 [50–65] % versus 58 [46–63] %; *P*=0.03) compared with patients without mPPM. No difference was noted in the rates of pre- procedural low gradient AS in patients with and without moderate-to-severe mPPM (20.6% versus 19.1%; *P*=0.75). Both measured and predicted moderate-to-severe PPM were more frequent following BEV than SEV (Table 3). SEV were used in smaller yet less calcified valves (Table 3). SEVs were associated with significantly larger measured and predicted EOA/indexed EOA and lower mean gradients, although the proportion of patients with a mean gradient >20 mm Hg did not differ between groups. Severe mPPM remained more prevalent in the BEV cohort, whereas severe pPPM was rare across both valve types without a significant intergroup difference (Table 3). When the projected EOA of the fifth-generation BEV was applied, the incidence of pPPM was substantially reduced in both the overall cohort (14% versus 0.5%; *P*<0.001) and the small-annulus subgroup (38% versus 1.9%; *P*<0.001; Figure S1).

**Table 3.**
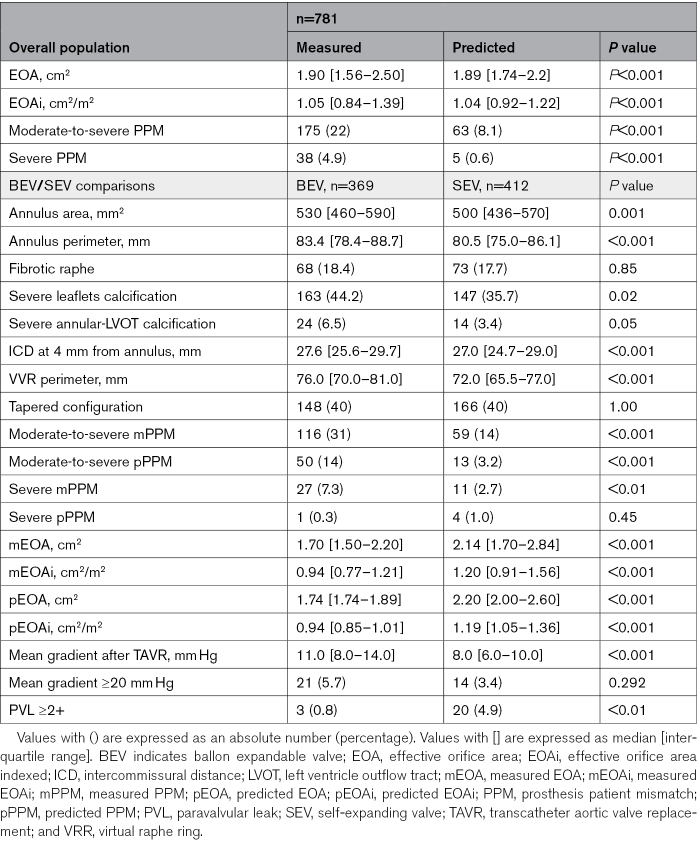
Differences in EOA and PPM According to Measured Versus Predicted Values and BEV Versus SEV Platforms

### Predictors of PPM

Univariate predictors of PPM are detailed in Tables S2 and S3. Neither leaflets nor left ventricular outflow tract/annular calcification, annular or supra-annular dimensions, nor a tapered aortic configuration were associated with the occurrence of mPPM (Table S2). On multivariable analysis, BEV and the absence of balloon predilatation were independently associated with mPPM (Table 4). All annular and supra-annular dimensions were associated with pPPM. As per mPPM, neither leaflets or left ventricular outflow tract/annular calcification nor a tapered aortic configuration were linked to pPPM (Table S3). Independent predictors of pPPM included smaller intercommissural distance at 4 mm above the annulus, THV type, small valve size, and a supra-annular sizing strategy (Table 4).

**Table 4.**
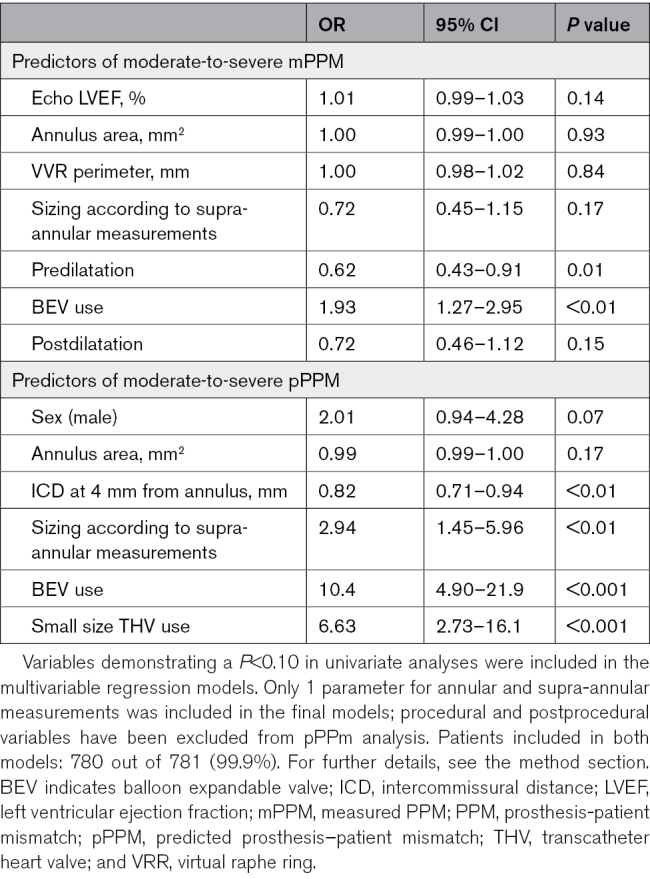
Multivariable Analysis for Predictors of Measured and Predicted PPM

### PPM and Outcomes

**Figure 3.**
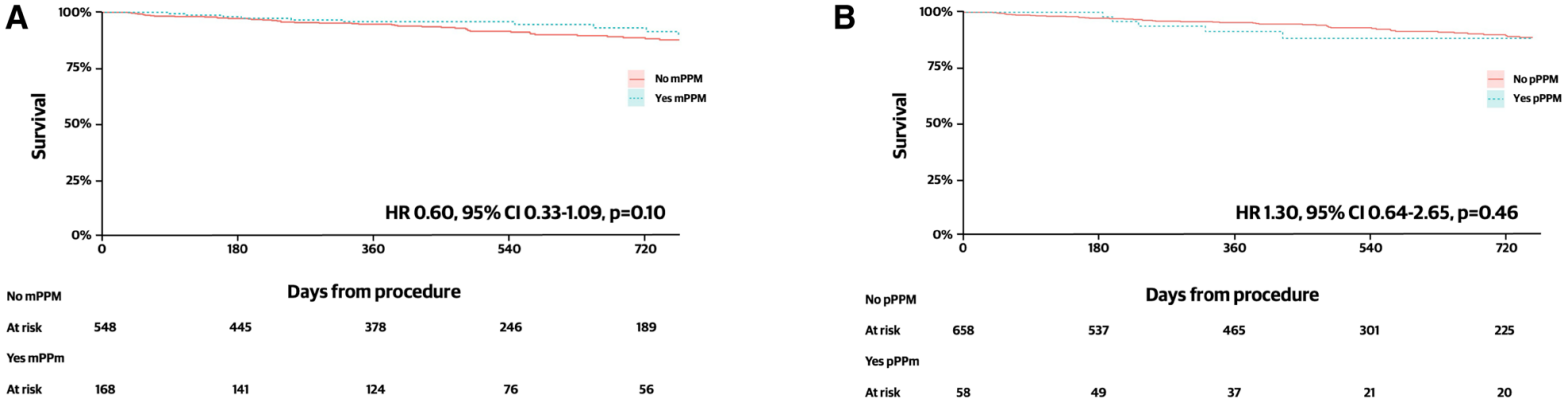
**Kaplan-Meier analysis for all-cause mortality according to measured prosthesis-patient mismatch (mPPM) and predicted prosthesis-patient mismatch (pPPM).** Kaplan-Meier curve and Cox estimates for mPPM (**A**). Kaplan-Meier curve and Cox estimates for pPPM (**B**). HR indicates hazard ratio.

Post-procedural follow-up (median, 524 days) was available for 716 patients, during which 79 (11%) met the primary outcome. Overall survival at 1-year was 95% (Figure 3). Moderate-to-severe mPPM was not associated with adverse events, nor was moderate-to-severe pPPM in the overall cohort (Figure 3). Likewise, neither mPPM (HR, 0.89 [95% CI, 0.38–2.08];*P*=0.79) nor pPPM (HR, 1.50 [95% CI, 0.50–4.46];*P*=0.47) was associated with cardiovascular mortality. Additional secondary outcomes—including hospitalization for heart failure, BVF, and transient ischemic attack or stroke—occurred in 19 (2.7%), 17 (2.4%), and 11 (1.5%) patients, respectively. No associations were observed between either mPPM or pPPM and these secondary outcomes (Table S4).

### Small Aortic Annuli Subgroup

**Figure 4.**
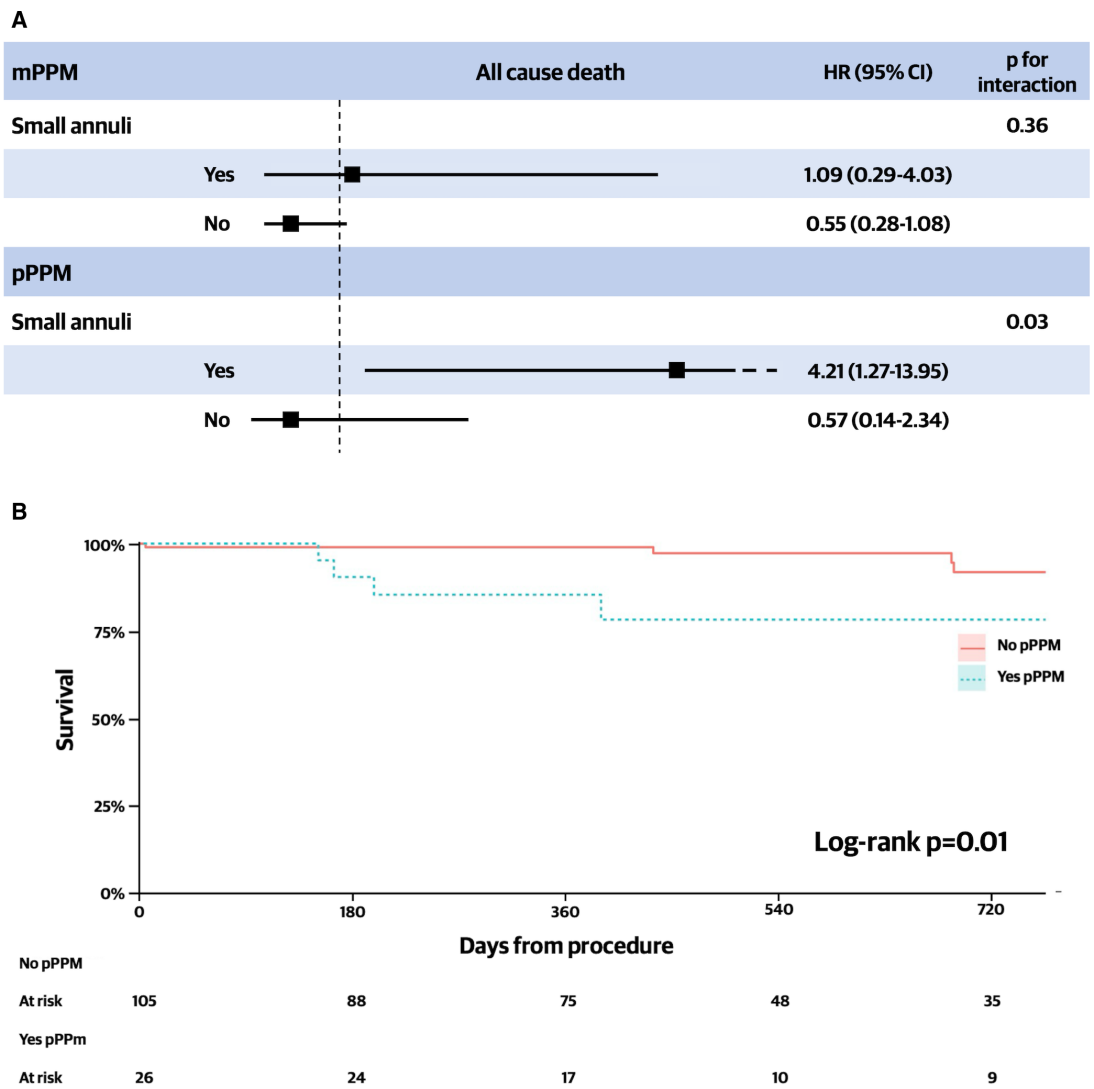
**All-cause mortality according to prosthesis-patient mismatch (PPM) in small and large annuli.** All-cause death forest plot for measured PPM (mPPM) and predicted PPM (pPPM) according to annular size (**A**). Kaplan-Meier analysis for all-cause mortality according to pPPM in small annuli (**B**). HR indicates hazard ratio.

In the small-annulus subgroup (n=145), both annular and supra-annular dimensions were significantly smaller than in patients with larger annuli (Table S5). The incidence of pPPM was higher in this subgroup (19% versus 5.5%; *P*<0.001), whereas rates of mPPM were comparable between small and large annuli (21% versus 23%; *P*=0.83) Table 5 . The distribution of annular size, use of small THVs, and PPM status is detailed in Figure S2. In exploratory survival analyses, moderate-to-severe pPPM—but not mPPM— was associated with increased all-cause mortality, with a significant interaction between pPPM and annular dimensions (Figure 4). This association did not extend to cardiovascular mortality (HR, 3.21 [95% CI, 0.63–16.35];*P*=0.16 for pPPM; and HR, 1.36 [95% CI, 0.26–7.01]; *P*=0.72 for mPPM), likely reflecting the limited number of events (12 all-cause and 7 cardiovascular deaths, respectively).

## Discussion

In this international, multicenter cohort of Sievers type 1 BAV patients undergoing TAVR, we provide the first simultaneous evaluation of mPPM and pPPM. We found that pPPM is considerably less common than mPPM, that balloon predilatation and THV selection differentially influence each mismatch metric, and that only pPPM portends worse survival in the high-risk subgroup with small annuli (Table 5).

**Table 5.**
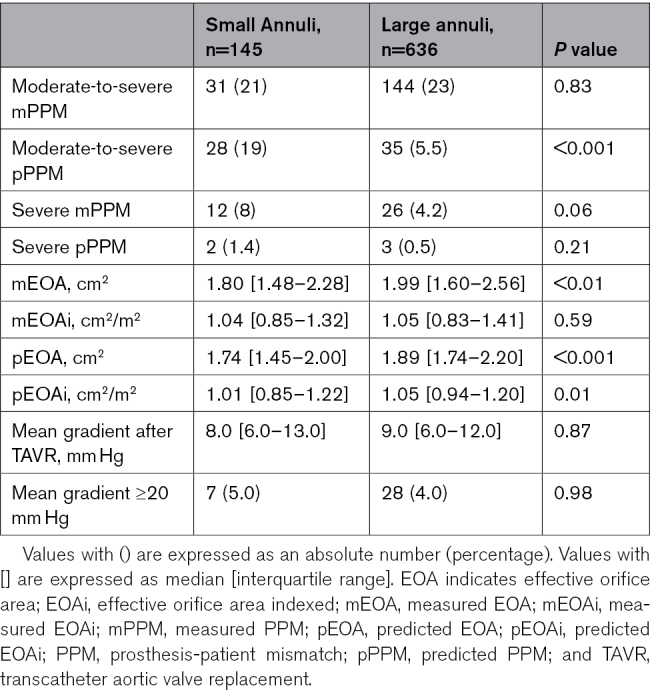
Differences in EOA and PPM According to Small Versus Large Annular Area

### Incidence of mPPM versus pPPM

Consistent with the tricuspid valve series, mEOA exhibited greater variability than pEOA, leading to a higher rate of mPPM.^11,17,18^ Unlike prior reports of lower mEOA versus pEOA, our BAV cohort demonstrated slightly larger mEOAs, likely reflecting unique bicuspid flow dynamics and pressure-recovery phenomena.^11,17,18,33,34^ This variability disproportionately places patients below Valve Academic Research Consortium-3 thresholds when relying on measured values, inflating mPPM estimates. However, in light of ongoing improvements in THV hemodynamics, the broader adoption of self-expanding platforms, and the increasing use of supra-annular sizing strategies, the incidence of PPM is likely to evolve. Notably, the fifth-generation SAPIEN BEV has already demonstrated superior hemodynamic performance in real-world practice.^32^ In line with these observations, our predictive analyses suggest that such advancements may translate into a substantially lower incidence of pPPM among patients with BAV anatomy (Figure S1). This finding is particularly relevant for patients at heightened risk of pPPM or its clinical consequences, including those undergoing intentional valve downsizing or those with small annular dimensions. Nonetheless, these results should be regarded as exploratory, and confirmation through large real-world data, together with direct measurement of PPM, will be essential.

### Predictors of PPM

BEV emerged as an independent risk factor for mPPM, whereas SEV and routine balloon predilatation were protective, underscoring the value of supra-annular leaflet position and meticulous valve predilatation in optimizing orifice expansion.^17,20,22,33,35,36^ By contrast, pPPM was driven almost entirely by preprocedural multislice computed tomography anatomy and sizing strategy: a narrower intercommissural distance, use of BEVs, smaller THV sizes, and supra-annular sizing each increased predicted mismatch. In essence, the anatomic constraints and the intentional down-sizing strategies sometimes necessary in tapered aortic roots are the primary determinants of whether a valve will be undersized for a given annulus.

### Prognostic Impact

In the overall cohort, neither measured nor pPPM was associated with mortality, consistent with prior multicenter TAVR registries that have reported no significant impact of PPM on survival in unselected populations.^11,33^ In contrast, within the subgroup of patients with small annuli, moderate-to-severe pPPM—but not mPPM—was associated with higher all-cause mortality, although the association did not extend to cardiovascular mortality. Previous studies reported that severe, but not moderate, mPPM was associated with increased all-cause and cardiovascular mortality, as well as higher rates of heart failure hospitalization, in populations characterized by predominantly small annuli and frequent implantation of small THVs.^12^ Our small-annulus cohort demonstrated comparable annular dimensions and THV sizes, and moderate-to-severe pPPM, but not mPPM, was independently associated with higher all-cause mortality. Notably, the moderate-to-severe mPPM group in our study primarily consisted of moderate cases. Therefore, it is unsurprising that mPPM did not influence mortality, even among patients with small annuli. Unlike mPPM, which may vary with hemodynamic conditions and can be affected by echocardiographic overestimation of transvalvular gradients, pPPM reflects prosthesis-specific effective orifice characteristics and remains stable across different flow states.^13–15^ This flow-independent limitation, even when moderate, may be particularly detrimental in the setting of small annuli requiring implantation of undersized prostheses. This parallel surgical series in which small bioprostheses and the associated pPPM have been linked to adverse outcomes.^9,37^ Although these findings suggest that anatomic constraints in small annuli may contribute to reduced EOA and potentially influence long-term outcomes, the small subset of patients and the limited number of events preclude definitive conclusions, and the results should be considered exploratory and hypothesis-generating. Further prospective studies with larger populations, standardized imaging, and detailed hemodynamic assessment are needed to clarify the interplay between anatomic restriction, valve design, and postprocedural gradients in determining the prognostic impact of PPM after TAVR in bicuspid anatomy.

### Clinical Implications

These findings translate into actionable strategies for TAVR in BAV anatomies. First, routine balloon predilatation and consideration of SEV can minimize mPPM by optimizing annular expansion and leaflet opening. Second, precise multislice computed tomography-based sizing must directly inform prosthesis selection to avoid pPPM. In patients with small or tapered roots, appropriate supra-annular sizing algorithms or newer THV designs should be considered to reduce predicted mismatch. Finally, incorporation of pEOA into the preprocedural workflow may better identify anatomies at risk for true orifice limitation, enabling targeted surveillance and timely adjunctive interventions.

### Limitations

Several limitations should be acknowledged. First, the retrospective, observational design of the AD-HOC registry introduces the potential for selection bias and unmeasured confounding, which limits causal inference regarding PPM determinants and outcomes. Second, echocardiographic and CT data were analyzed at each site without core-laboratory adjudication, which may have introduced interobserver variability in orifice area measurements and annular sizing. Third, patients with small annuli represented a limited proportion of the cohort, and the number of clinical events was relatively low. For this reason, multivariable analysis was not performed, and, as a result, the observed association between pPPM and all-cause mortality should be interpreted as exploratory and hypothesis-generating only. Moreover, the absence of a corresponding association with cardiovascular mortality suggests that this relationship may be influenced by competing risks or residual confounders rather than a direct effect of PPM on cardiac outcomes. Fourth, procedural factors such as valve selection, sizing strategy, and use of predilatation or postdilatation were left to operator discretion and varied across centers, potentially confounding the observed associations. The small number of patients enrolled in some centers precluded clustering by center in the regression analyses. Therefore, potential center-level effects cannot be excluded and should be considered when interpreting the results. Although our multivariable model accounted for a comprehensive range of predictors, the influence of those unmeasured confounders cannot be completely excluded. Thus, further investigations are warranted to clarify the precise impact of sizing strategies and pre- and postdilatation on PPM occurrence. Additionally, variable selection for multivariable models partly relied on univariable screening using a *P*<0.10 threshold, potentially introducing bias by excluding potentially relevant variables. Therefore, even if the final models included all the variables with clinical relevance, the results should be interpreted in this regard. Finally, even though PPM exerted detrimental effects as early as 1 to 3 years after follow-up in prior studies, with a mean follow-up of 524 days, we cannot exclude the emergence of late valve degeneration or longer-term impacts of PPM on valve durability and clinical outcomes beyond the study period. Despite these limitations, the study’s multicenter design, rigorous statistical adjustment, and consistent findings across centers provide a robust framework for hypothesis generation.

### Conclusions

In patients with severe Sievers type 1 BAV undergoing TAVR, neither measured nor pPPM was associated with adverse clinical outcomes in the overall cohort. Among patients with small annuli, moderate-to-severe pPPM was associated with increased all-cause mortality; however, this association did not extend to cardiovascular mortality and should be interpreted as exploratory. In this anatomically constrained population, anatomic limitation—rather than prosthesis design—appears to be the predominant determinant of EOA. Collectively, these findings highlight the need for dedicated prospective studies to further elucidate the interplay among valve sizing, anatomic constraint, and long-term outcomes following TAVR in BAV anatomy.

## ARTICLE INFORMATION

### Disclosures

None.

### Supplemental Material

Tables S1–S5

Figures S1–S2

## Supplementary Material
