# Supplementary material for: Measured Versus Predicted Prosthesis–Patient Mismatch after TAVR in Sievers Type 1 BAV: Incidence, Determinants, and Outcomes From the AD-HOC Registry
Source: Circ Cardiovasc Interv. 2026 May 12;19(7):e015994. doi: 10.1161/CIRCINTERVENTIONS.125.015994 (PMC13384377; doi:10.1161/CIRCINTERVENTIONS.125.015994)
Supplement: Supplementary file 1 [file hcv-19-e015994-s001.pdf]

SUPPLEMENTAL MATERIAL

Measured vs. Predicted Prosthesis-Patient Mismatch after TAVR in Sievers Type 1 BAV: Incidence, Determinants, and Outcomes from the AD-HOC Registry

SUPPLEMENTAL FIGURES

Supplemental Figure 1: Rate of pPPM according to Sapien BEV generations.

Actual rates of pPPM of previous BEV generation (red boxes) vs. predicted pPPM rates in 5th BEV device (blue boxes) in overall and small annuli population and patients undergoing supraannular sizing.

BEV: ballon expandable valves; pPPM: predicted prosthesis-patient mismatch; S3/S3U: Sapien 3/Sapien 3 Ultra.

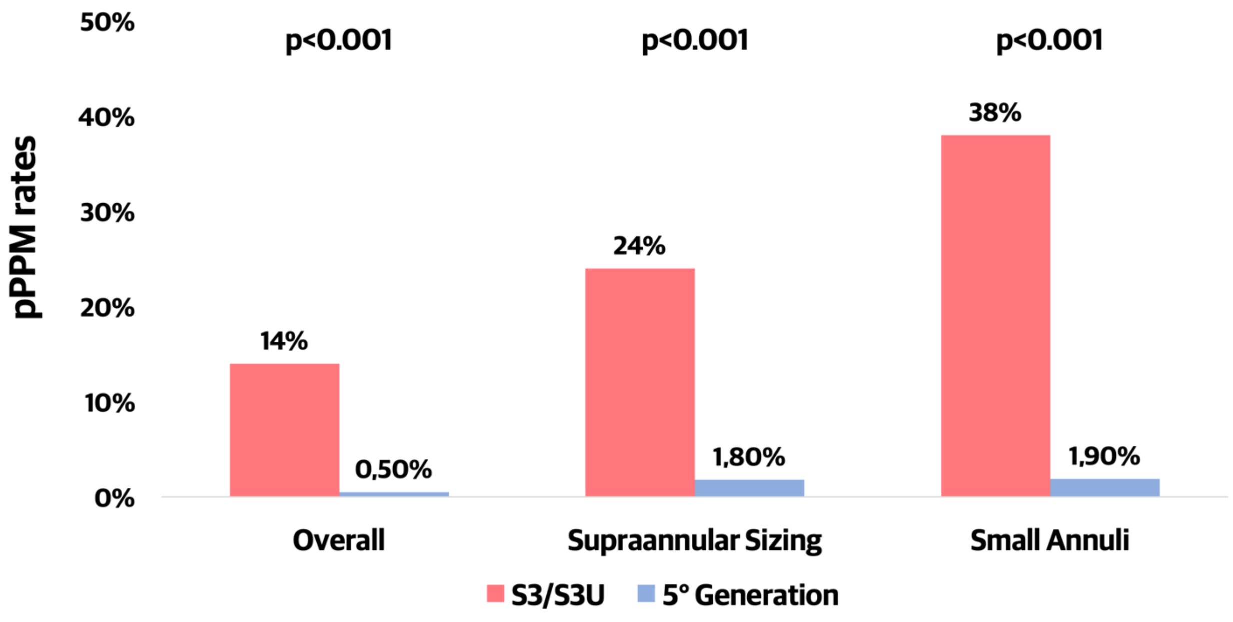

Supplemental Figure 2: Differences in EOA/EOAi and Small THV distribution according to annular size and mismatch classification.

Measured EOA according to annular size and mPPM (A). Measured EOAi according to annular size and mPPM (B). Small THV distribution according to annular size and mPPM (C). Predicted EOA according to annular size and pPPM (D). Predicted EOAi according to annular size and pPPM (E). Small THV distribution according to annular size and mPPM (F).

EOA: effective orifice area; EOAi: effective orifice area indexed; mPPM: measured PPM; PPM: prosthesis-patient mismatch; pPPM: predicted PPM; THV: transcatheter heart valve.

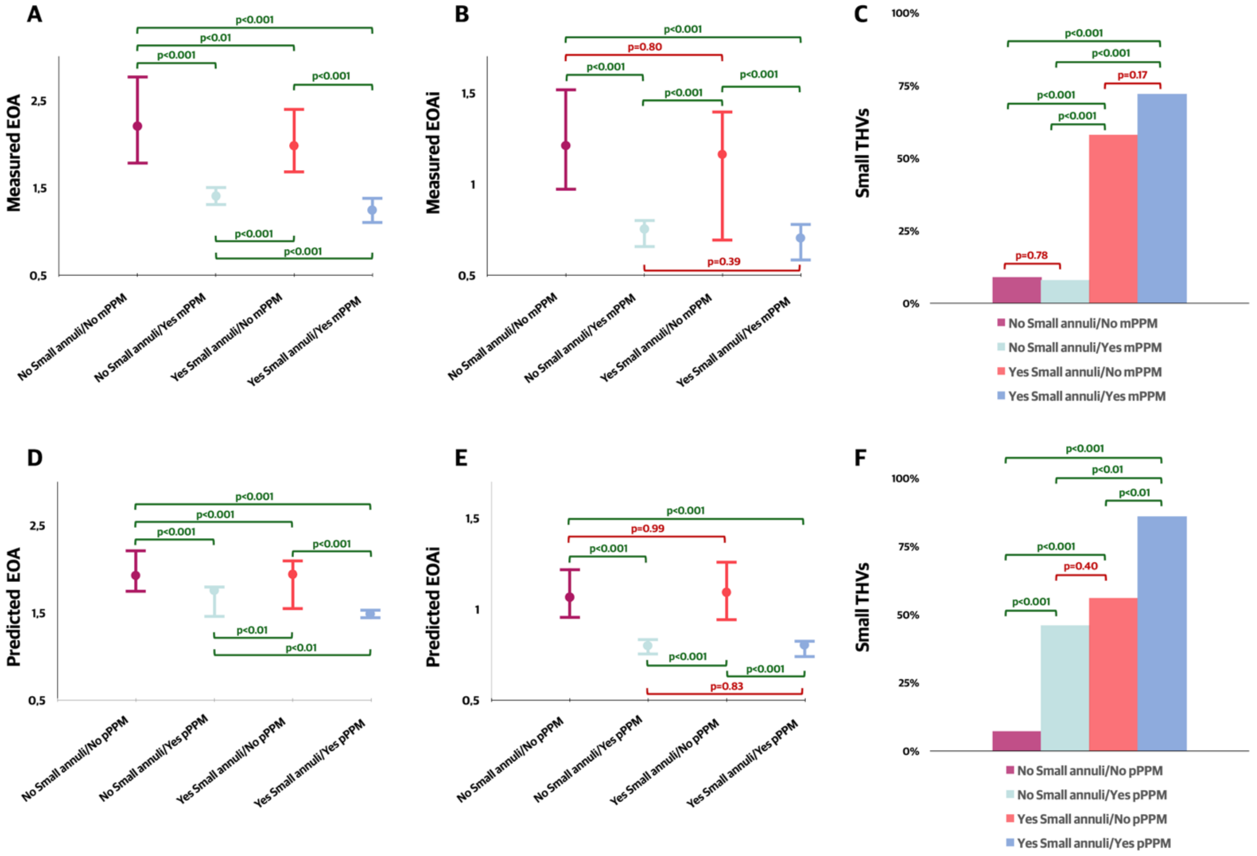

SUPPLEMENTAL TABLES

Supplemental Table 1: Platform and size of THV with relative PPM rates used in the study.

Values with ( ) are expressed as absolute number (percentage). Percentage in “n” columns refers to total study population, percentage in “mPPM” and “pPPM” columns refers to each specific valve type and size. **Size S, M and L refer to Acurate Neo/Neo2 platforms; PPM: prosthesis-patient mismatch; pPPM: predicted PPM; mPPM: measured PPM; THV: transcatheter heart valve.*

Cluster of THV	Size 20 mm			Size 23 mm/S*			Size 25 mm/M*			Size 26 mm			Size 27 mm/L*			Size 29 mm			Size 34 mm			Total (for each platform)		
	n	mPPM	pPPM	n	mPPM	pPPM	n	mPPM	pPPM	n	mPPM	pPPM	n	mPPM	pPPM	n	mPPM	pPPM	n	mPPM	pPPM	n	mPPM	pPPM
**Sapien 3**	0 (0)	0 (0)	0 (0)	27 (3.5)	13 (48)	11 (41)	-	-	-	69 (8.8)	26 (38)	10 (15)	-	-	-	122 (16)	38 (31)	2 (1.6)	-	-	-	218 (28)	77 (35)	23 (11)
**Sapien 3 U**	1 (0.1)	0 (0)	0 (0)	34 (4.4)	6 (17)	18 (53)	-	-	-	95 (12)	28 (30)	9 (9.5)	-	-	-	21 (2.7)	5 (24)	0 (0)	-	-	-	151 (19)	39 (26)	27 (18)
**Acurate Neo**	-	-	-	12 (1.5)	4 (33)	1 (8.3)	17 (2.1)	6 (35)	0 (0)	-	-	-	22 (2.8)	2 (9.1)	0 (0)	-	-	-	-	-	-	51 (6.5)	12 (24)	1 (2)
**Acurate Neo2**	-	-	-	8 (1.0)	2 (25)	0 (0)	10 (1.3)	2 (20)	0 (0)	-	-	-	12 (1.5)	4 (33)	0 (0)	-	-	-	-	-	-	30 (3.8)	8 (27)	0 (0)
**Evolut R**	-	-	-	7 (0.9)	1 (14)	7 (100)	-	-	-	23 (2.9)	6 (26)	3 (13)	-	-	-	30 (3.8)	4 (13)	0 (0)	85 (11)	12 (14)	0 (0)	145 (19)	23 (16)	10 (6.9)
**Evolut Pro**	-	-	-	0 (0)	0 (0)	0 (0)	-	-	-	22 (2.8)	2 (9.1)	0 (0)	-	-	-	71 (9.1)	5 (7)	1 (1.4)	-	-	-	93 (12)	7 (7)	1 (1)
**Evolut Pro +**	-	-	-	1 (0.1)	0 (0)	0 (0)	-	-	-	14 (1.8)	1 (7.1)	0 (0)	-	-	-	42 (5.4)	7 (17)	0 (0)	25 (3.2)	1 (4)	0 (0)	82 (10)	9 (11)	0 (0)
**Navitor**	-	-	-	0 (0)	0 (0)	0 (0)	2 (0.3)	0 (0)	0 (0)	-	-	-	5 (0.6)	0 (0.0)	1 (20)	4 (0.5)	0 (0)	0 (0)	-	-	-	11 (1.4)	0 (0)	1 (9.1)
**Total (for each size)**	1 (0.1)	0 (0)	0 (0)	89 (11)	26 (29)	37 (42)	29 (3.7)	8 (28)	0 (0)	223 (29)	63 (28)	22 (9.9)	39 (4.9)	6 (15)	1 (2.6)	290 (37)	59 (20)	3 (1.0)	110 (14)	13 (12)	0 (0)	781 (100)	175 (22)	63 (8.1)

Supplemental Table 2: Univariate analysis for predictors of measured PPM.

BEV: balloon expandable valve; ICD: intercommisural distance; LVEF: left ventricle ejection fraction; LVOT: left ventricle outflow tract; PPM: prosthesis-patient mismatch; mPPM: measured PPM; PVL: paravalvular regurgitation; THV: transcatheter heart valve; VRR: virtual raphe ring.

**Predictors of moderate-to-severe mPPM**			
	OR	***95% CI***	***p***
Sex (Male)	1.22	0.85, 1.73	0.29
Echo LVEF (%)	1.01	0.99, 1.02	0.08
Annulus area (mm^2)	1.00	0.99, 1.00	0.73
Annulus perimeter (mm)	0.99	0.97, 1.01	0.59
Aortic Angle (°)	1.01	0.99, 1.02	0.36
Severe leaflets calcification	1.22	0.86, 1.73	0.26
Severe annular-LVOT calcification	1.25	0.60, 2.63	0.55
Fibrotic Raphe	1.29	0.85, 1.97	0.23
VVR perimeter (mm)	1.02	0.99, 1.04	0.09
ICD at VRR (mm)	0.99	0.94, 1.04	0.60
ICD at 4 mm from annulus (mm)	0.97	0.93, 1.03	0.42
CASPER diameter (mm)	1.01	0.97, 1.05	0.71
Tapered configuration	0.87	0.62, 1.24	0.45
Sizing according to supra-annular measurements	0.68	0.43, 1.06	0.09
**BEV use**	**2.74**	**1.92, 3.90**	**<0.001**
**Pre-dilatation**	**0.46**	**0.33, 0.65**	**<0.001**
Small size THV use	1.08	0.71, 1.65	0.73
Supra-annular landing	0.61	0.23, 1.38	0.23
**Post-dilatation**	**0.44**	**0.30, 0.66**	**<0.001**
Moderate-severe THV constriction	0.73	0.35, 1.53	0.40
Supra-annular constriction	1.30	0.66, 2.57	0.45
PVL ≥ 2+	0.51	0.15, 1.74	0.28

Supplemental Table 3: Univariate analysis for predictors of predicted PPM.

BEV: balloon expandable valve; ICD: intercommisural distance; LVEF: left ventricle ejection fraction; LVOT: left ventricle outflow tract; PPM: prosthesis-patient mismatch; pPPM: predicted PPM; THV: transcatheter heart valve; VRR: virtual raphe ring.

**Predictors of moderate-to-severe pPPM**			
	OR	***95% CI***	***p***
**Sex (Male)**	**0.49**	**0.29, 0.82**	**<0.01**
Echo LVEF (%)	1.00	0.98, 1.03	0.66
**Annulus area (mm^2)**	**0.99**	**0.99, 0.99**	**<0.001**
**Annulus perimeter (mm)**	**0,89**	**0,86, 0,92**	**<0.001**
Aortic Angle (°)	0.98	0.96, 1.00	0.17
Severe leaflets calcification	1.58	0.90, 2.77	0.11
Severe annular-LVOT calcification	1.79	0.67, 4.75	0.25
Fibrotic Raphe	1.32	0.71, 2.48	0.37
**VVR perimeter (mm)**	**0.94**	**0.91, 0.96**	**<0.001**
**ICD at VVR (mm)**	**0,79**	**0.73, 0.85**	**<0.001**
**ICD at 4 mm from annulus (mm)**	**0.76**	**0.69, 0.83**	**<0.001**
**CASPER diameter (mm)**	**0.78**	**0.71, 0.86**	**<0.001**
Tapered configuration	1.20	0.72, 2.03	0.48
**Sizing according to supra-annular measurements**	**2.23**	**1.29, 3.87**	**<0.01**
**BEV use**	**4.83**	**2.58, 9.04**	**<0.001**
**Small size THV use**	**9.80**	**5.64, 17.04**	**<0.001**

Supplemental Table 4: Cox regression analysis for secondary outcomes.

For each outcomes no mPPM or pPPM has been chosen as reference level.

BVD: bioprosthetic valve disfunction; PPM: prosthesis patient mismatch; mPPM: measured PPM; pPPM: predicted PPM.

**mPPM**			
	**HR**	**95% CI**	**p**
Heart failure hospitalization	0.35	0.08-1.52	p=0.16
TIA/Stroke	1.04	0.27-4.03	p=0.96
BVD	0.19	0.03-1.43	p=0.11
**pPPM**			
	**HR**	**95% CI**	**p**
Heart failure hospitalization	1.30	0.30-5.66	p=0.72
TIA/Stroke	1.79	0.37-8.74	p=0.47
BVD	2.50	0.72-8.73	p=0.15

Supplemental Table 5: Relevant characteristics according to small vs large annuli.

Values with ( ) are expressed as absolute number (percentage). Values with [ ] are expressed as median [interquartile range].

AV: aortic valve; BEV: balloon expandable valve; BMI: body mass index; BSA: body surface area; eGFR: estimated glomerular filtration rate; ICD: intercommissural distance; LVEF: left ventricle ejection fraction; STS-PROM: Society of Thoracic Surgeons predicted risk of mortality; THV: transcatheter heart valve; VRR: virtual raphe ring.

**Population characteristics**	**Small Annuli n 145**	**Large annuli n 636**	**p-value**
Age	79 [75, 83]	78 [73, 83]	0.23
BMI (kg/m2)	24.6 [22.2, 27.3]	25.3 [23.0, 28.7]	<0.01
BSA (m2)	1.7 [1.6, 1.9]	1.9 [1.7, 2.0]	<0.001
STS-PROM score (%)	3.10 [2.18, 4.40]	2.30 [1.40, 3.50]	<0.001
Sex (Male)	46 (32)	450 (71)	<0.001
History of Atrial fibrillation	34 (24)	179 (28)	0.30
eGFR (ml/min)	66 [51, 84]	67 [53, 84]	0.48
Echo LVEF (%)	60 [50, 65]	58 [45, 64]	0.04
AV mean gradient (mmHg)	50 [40, 60]	47 [40, 57]	0.06
Pre-dilatation	103 (71)	414 (65)	0.20
Sizing according to supra-annular measurements	27 (18)	134 (21)	0.57
BEV use	53 (37)	316 (50)	<0.01
Small THV use	90 (62)	59 (9)	<0.001
Post-dilatation	55 (38)	215 (34)	0.38
Annulus area (mm^2)	391 [357, 416]	539 [488, 596]	<0.001
Annulus perimeter (mm)	73 [69, 75]	83 [79, 88]	<0.001
Aortic angle (°)	51 [44, 59]	53 [46, 61]	0.03
Leaflets calcification			<0.01
Moderate	70 (48)	262 (41)	
Severe	41 (28.3)	269 (42)	
Fibrotic Raphe	42 (29)	99 (16)	<0.001
VRR perimeter (mm)	67 [62, 71]	75 [70, 80]	<0.001
ICD at VVR (mm)	24.0 [22.7, 26.0]	27.7 [26.0, 29.6]	<0.001
ICD at 4 mm (mm)	24.0 [22.7, 25.9]	27.6 [26.0, 29.5]	<0.001
CASPER diameter (mm)	21.8 [20.5, 23.1]	25.0 [23.3, 26.6]	<0.001
Tapered configuration	62 (43)	252 (40)	0.51
